# Tuberculosis — United States, 2020

**DOI:** 10.15585/mmwr.mm7012a1

**Published:** 2021-03-26

**Authors:** Molly Deutsch-Feldman, Robert H. Pratt, Sandy F. Price, Clarisse A. Tsang, Julie L. Self

**Affiliations:** ^1^Epidemic Intelligence Service, CDC; ^2^Division of Tuberculosis Elimination, National Center for HIV/AIDS, Viral Hepatitis, STD, and TB Prevention, CDC.

Tuberculosis (TB) disease incidence has decreased steadily since 1993 (*1*), a result of decades of work by local TB programs to detect, treat, and prevent TB disease and transmission. During 2020, a total of 7,163 TB cases were provisionally reported to CDC’s National Tuberculosis Surveillance System (NTSS) by the 50 U.S. states and the District of Columbia (DC), a relative reduction of 20%, compared with the number of cases reported during 2019.* TB incidence per 100,000 persons was 2.2 during 2020, compared with 2.7 during 2019. Since 2010, TB incidence has decreased by an average of 2%–3% annually (*1*). Pandemic mitigation efforts and reduced travel might have contributed to the reported decrease. The magnitude and breadth of the decrease suggest potentially missed or delayed TB diagnoses. Health care providers should consider TB disease when evaluating patients with signs and symptoms consistent with TB (e.g., cough of >2 weeks in duration, unintentional weight loss, and hemoptysis), especially when diagnostic tests are negative for SARS-CoV-2, the virus that causes COVID-19. In addition, members of the public should be encouraged to follow up with their health care providers for any respiratory illness that persists or returns after initial treatment. The steep, unexpected decline in TB cases raises concerns of missed cases, and further work is in progress to better understand factors associated with the decline.

Health departments in the 50 U.S. states and DC report cases of TB to CDC based on the Council for State and Territorial Epidemiologists’ surveillance definition, which includes both laboratory and clinically verified cases.^†^ For each case, health departments electronically submit a report of a verified case of TB to CDC. Although certain jurisdictions reported disruptions to routine TB prevention activities early in the pandemic (*2*), all reporting areas provided provisional reporting data to CDC. Among these reports, <5% of the data were missing, providing further confidence that they were reasonably complete. Provisional data were used to calculate national- and state-level TB case counts. Midyear U.S. Census Bureau population estimates^§^ were used for calculating national- and state-level TB incidence per 100,000 persons. Case reports were grouped on the basis of self-reported race and ethnicity according to federal guidelines.^¶^ Persons self-reporting Hispanic ethnicity are categorized as Hispanic regardless of self-reported race, persons not reporting Hispanic ethnicity are categorized by self-reported race, and non-Hispanic persons who self-reported more than one race are categorized as “multiple races.” Midyear population estimates from the Current Population Survey** were used to calculate incidence by national origin and race/ethnicity.

A total of 7,163 TB cases were reported during 2020 (2.2 cases per 100,000 persons), 20% fewer than during 2019 (2.7 cases per 100,000 persons). Thirty-nine states and DC reported a decrease in cases, eight states reported an increase, and three reported no change. California reported the highest number of cases (1,703), and Alaska reported the highest incidence (7.9 cases per 100,000 persons) (Table 1). The East North Central region experienced the largest decrease in TB incidence (−25%).

**TABLE 1:** Tuberculosis (TB) disease case counts, incidence, and annual percentage changes, by U.S. Census division and state — 50 states and the District of Columbia, 2019–2020

U.S. Census division	No. of cases*			TB incidence^†^		
	2019	2020	% Change	2019	2020	% Change^§^
**Division 1: New England**						
Connecticut	67	54	–19.4	1.9	1.5	–19.2
Maine	18	17	–5.6	1.3	1.3	–5.9
Massachusetts	178	142	–20.2	2.6	2.1	–20.2
New Hampshire	6	12	100.0	0.4	0.9	99.2
Rhode Island	14	9	–35.7	1.3	0.9	–35.7
Vermont	4	3	–25.0	0.6	0.5	–24.9
**Subtotal**	**287**	**237**	**–17.4**	**1.9**	**1.6**	**–17.4**
**Division 2: Middle Atlantic**						
New Jersey	310	237	–23.5	3.5	2.7	–23.5
New York	746	606	–18.8	3.8	3.1	–18.2
Pennsylvania	198	158	–20.2	1.5	1.2	–20.1
**Subtotal**	**1,254**	**1,001**	**–20.2**	**3.0**	**2.4**	**–19.9**
**Division 3: East North Central**						
Illinois	326	216	–33.7	2.6	1.7	–33.3
Indiana	108	92	–14.8	1.6	1.4	–15.1
Michigan	131	101	–22.9	1.3	1.0	–22.8
Ohio	150	130	–13.3	1.3	1.1	–13.3
Wisconsin	51	34	–33.3	0.9	0.6	–33.4
**Subtotal**	**766**	**573**	**–25.2**	**1.6**	**1.2**	**–25.1**
**Division 4: West North Central**						
Iowa	52	39	–25.0	1.6	1.2	–25.1
Kansas	38	38	—	1.3	1.3	—
Minnesota	148	117	–20.9	2.6	2.1	–21.2
Missouri	70	68	–2.9	1.1	1.1	–3.0
Nebraska	17	36	111.8	0.9	1.9	111.2
North Dakota	18	10	–44.4	2.4	1.3	–44.6
South Dakota	16	16	—	1.8	1.8	–0.6
**Subtotal**	**359**	**324**	**–9.7**	**1.7**	**1.5**	**–9.9**
**Division 5: South Atlantic**						
Delaware	19	16	–15.8	1.9	1.6	–16.7
District of Columbia	24	19	–20.8	3.4	2.7	–21.3
Florida	558	413	–26.0	2.6	1.9	–26.8
Georgia	298	221	–25.8	2.8	2.1	–26.4
Maryland	209	147	–29.7	3.5	2.4	–29.7
North Carolina	185	158	–14.6	1.8	1.5	–15.4
South Carolina	80	67	–16.3	1.6	1.3	–17.2
Virginia	191	168	–12.0	2.2	2.0	–12.4
West Virginia	10	13	30.0	0.6	0.7	30.8
**Subtotal**	**1,574**	**1,222**	**–22.4**	**2.4**	**1.8**	**–23.0**
**Division 6: East South Central**						
Alabama	87	75	–13.8	1.8	1.5	–14.0
Kentucky	66	67	1.5	1.5	1.5	1.4
Mississippi	58	41	–29.3	1.9	1.4	–29.0
Tennessee	129	113	–12.4	1.9	1.6	–13.1
**Subtotal**	**340**	**296**	**–12.9**	**1.8**	**1.5**	**–13.2**
**Division 7: West South Central**						
Arkansas	64	59	–7.8	2.1	1.9	–8.1
Louisiana	88	99	12.5	1.9	2.1	12.8
Oklahoma	73	67	–8.2	1.8	1.7	–8.7
Texas	1,162	888	–23.6	4.0	3.0	–24.6
**Subtotal**	**1,387**	**1,113**	**–19.8**	**3.4**	**2.7**	**–20.5**
**Division 8: Mountain**						
Arizona	183	136	–25.7	2.5	1.8	–27.0
Colorado	66	52	–21.2	1.1	0.9	–21.9
Idaho	7	8	14.3	0.4	0.4	11.9
Montana	2	4	100.0	0.2	0.4	98.1
Nevada	53	57	7.5	1.7	1.8	5.9
New Mexico	41	30	–26.8	2.0	1.4	–27.1
Utah	27	25	–7.4	0.8	0.8	–8.7
Wyoming	1	0	–100.0	0.2	—	–100.0
**Subtotal**	**380**	**312**	**–17.9**	**1.5**	**1.2**	**–19.0**
**Division 9: Pacific**						
Alaska	58	58	—	7.9	7.9	0.3
California	2,114	1,703	–19.4	5.4	4.3	–19.3
Hawaii	99	92	–7.1	7.0	6.5	–6.5
Oregon	70	67	–4.3	1.7	1.6	–4.9
Washington	221	165	–25.3	2.9	2.1	–26.1
**Subtotal**	**2,562**	**2,085**	**–18.6**	**4.8**	**3.9**	**–18.7**
**Total**	**8,909**	**7,163**	**–19.6**	**2.7**	**2.2**	**–19.9**

* Based on data reported to National Tuberculosis Surveillance System as of February 17, 2021.

^†^ Cases per 100,000 persons. Rates calculated by using midyear population estimates from the U.S. Census Bureau.

^§^ Calculated by using unrounded figures.

During 2020, 71% of TB cases occurred among non–U.S.-born^††^ persons, the same proportion as in 2019. Incidence decreased among both U.S.-born (0.9 to 0.7 cases per 100,000 persons) and non–U.S.-born persons (14.2 to 11.5 cases per 100,000 persons) (Figure). Among U.S.-born persons reported as having TB disease, 36% identified as Black, 28% as White, 24% as Hispanic, 5% as Asian, 4% as American Indian/Alaska Native (AI/AN), 2% as Native Hawaiian/other Pacific Islander (NH/PI), and 1% as multiple races.^§§^ TB incidence decreased among all U.S.-born groups, except NH/PI^¶¶^ (Table 2). Among non–U.S.-born persons with a diagnosis of TB, 48% identified as Asian, 32% as Hispanic, 13% as Black, 4% as White, 1% as NH/PI, 1% as multiple races, and <1% as AI/AN. During both 2019 and 2020, the most frequently reported countries of birth among non–U.S.-born persons were Mexico, the Philippines, India, Vietnam, and China.

**FIGURE:**
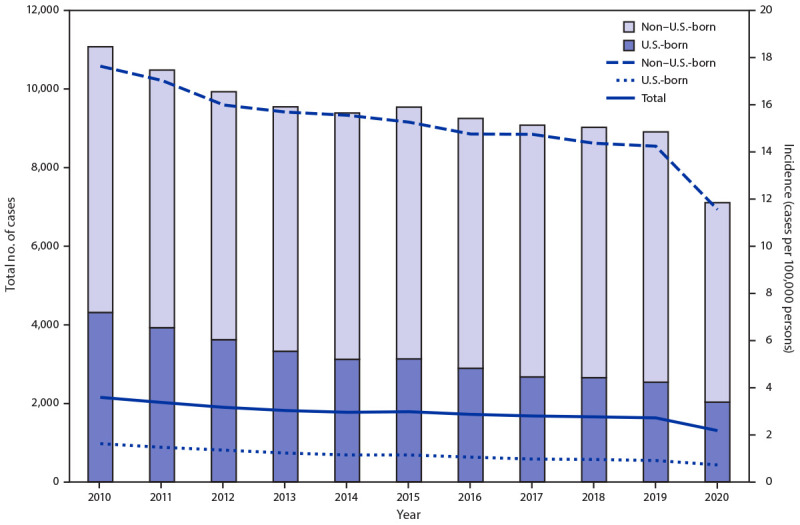
Tuberculosis disease cases and incidence, by birth origin*^,†^ — United States, 2010–2020 * Numbers of tuberculosis cases among persons with unknown origin are not shown (range = 2–61). Total rate includes cases among persons with unknown national origin. ^†^ Rates for non–U.S.-born and U.S.-born persons were calculated by using midyear Current Population Survey estimates. Total rate was calculated by using midyear population estimates from the U.S. Census Bureau.

**TABLE 2:** Tuberculosis disease case numbers and incidence per 100,000 persons, by race/ethnicity and birth origin — United States, 2017–2020

Birth origin and race/ethnicity	No. of cases* (incidence^†^)			
	2017	2018	2019	2020
**U.S.-born** ^§^				
Hispanic	582 (1.5)	585 (1.5)	611 (1.5)	485 (1.2)
White, non-Hispanic	794 (0.4)	809 (0.4)	762 (0.4)	569 (0.3)
Black, non-Hispanic	1,004 (2.8)	950 (2.7)	908 (2.6)	719 (2.0)
Asian	126 (1.8)	134 (1.9)	117 (1.5)	106 (1.3)
American Indian/Alaska Native	91 (3.8)	102 (4.0)	79 (3.4)	71 (3.2)
Native Hawaiian/other Pacific Islander	44 (6.4)	41 (5.4)	25 (3.8)	42 (6.2)
Multiple or unknown race/ethnicity	29 (—^¶^)	30 (—^¶^)	32 (—^¶^)	35 (—^¶^)
**Subtotal**	**2,670 (1.0)**	**2,651(1.0)**	**2,534 (0.9)**	**2,027 (0.7)**
**Non–U.S.-born**				
Hispanic	1,975 (10.0)	2,045 (10.3)	2,079 (10.3)	1,619 (8.0)
White, non-Hispanic	264 (3.4)	258 (3.2)	252 (3.1)	220 (2.8)
Black, non-Hispanic	903 (22.3)	844 (20.3)	837 (19.8)	662 (15.3)
Asian	3,136 (27.4)	3,072 (26.1)	3,043 (26.1)	2,422 (21.7)
American Indian/Alaska Native	2 (2.9)	2 (3.5)	2 (3.5)	1 (2.5)
Native Hawaiian/Pacific Islander	67 (22.7)	73 (24.7)	80 (24.8)	69 (32.5)
Multiple or unknown race/ethnicity	56 (—^¶^)	71 (—^¶^)	76 (—^¶^)	82 (—^¶^)
**Subtotal**	**6,403 (14.7)**	**6,365 (14.4)**	**6,369 (14.2)**	**5,075 (11.5)**
Unknown national origin	6	3	6	61
**Total**	**9,079 (2.8)**	**9,019 (2.8)**	**8,909 (2.7)**	**7,163 (2.2)**

* Based on data reported to National Tuberculosis Surveillance System as of February 17, 2021.

^†^ Cases per 100,000 persons. Rates for non–U.S.-born and U.S.-born persons were calculated by using Current Population Survey estimates. Total rate was calculated by using midyear population estimates from the U.S. Census Bureau.

^§^ A person is considered U.S.-born if eligible for U.S. citizenship at birth, regardless of place of birth.

^¶^ Rates could not be calculated for these categories because population estimates are not available.

During 2020, among all non–U.S.-born persons with TB cases, 10% had received a diagnosis ≤1 year after the person’s arrival in the United States, compared with an average of 16% during 2015–2019. In addition, the proportion of cases identified among non–U.S.-born persons living in the United States for >20 years increased to 32% from an average of 28% during 2015–2019. The age distribution of persons with TB cases during 2020 was similar to the average distribution during 2015–2019. The largest proportion of cases occurred among persons aged 45–64 years (30%), followed by those aged 25–44 years (29%), ≥65 years (26%), 15–24 years (10%), 5–14 years (2%), and ≤4 years (2%).

## Discussion

TB cases and incidence have decreased gradually since the peak of resurgence in 1992 (*1*), highlighting the impact of nationwide TB control efforts. Although steep decreases have been reported previously, most notably after the 2008 economic recession (*3*), the annual decrease reported during 2020 is far larger than any reported during the last decade (*1*). Similar trends in TB have been reported globally (*4*) and for other diseases domestically (*5*,*6*). Multiple factors have likely led to both a true decrease in TB incidence and underascertainment of cases.

The reduction in the number of persons with TB disease reported ≤1 year after arrival in the United States indicates that changes in immigration and travel patterns during 2020 might have contributed to a decrease in TB incidence. However, given the large proportion of cases that occur each year among persons who have been in the United States >1 year, particularly those who have been in the United States >10 years (*7*), and the broad decreases reported among both non–U.S.-born and U.S.-born populations, immigration and travel changes cannot fully explain the decrease in the number of reported TB cases during 2020. Another possible cause of this decrease is that mitigation strategies implemented for slowing the spread of COVID-19 (e.g., mask-wearing and social distancing) might have also reduced TB transmission.

The unexpectedly steep and widespread reduction in the number of reported TB cases causes concern regarding underdiagnosis. CDC has received anecdotal reports of persons who repeatedly sought medical attention for persistent TB signs and symptoms, received a negative test result for SARS-CoV-2 multiple times, and received a TB diagnosis much later (in certain cases on autopsy), demonstrating that other TB cases might have been missed during 2020. TB should be considered in the differential diagnosis of patients with prolonged (>2 weeks) cough or TB symptoms such as unintentional weight loss, particularly in the context of negative tests for SARS-CoV-2 and epidemiologic risk factors for TB (e.g., birth or former residence in a country with high TB incidence, a history of living in a congregate setting such as a homeless shelter or a correctional facility, or immune suppression). In such cases, health care providers should consider ordering rapid TB diagnostic tests (e.g., sputum microscopy or nucleic acid amplification tests) to quickly identify patients with TB disease. Clinical consultation for potential TB cases is also available through TB programs or the CDC-sponsored TB Centers of Excellence.***

Limited access to and reluctance to seek medical care during the COVID-19 pandemic have been reported (*8*) and might also contribute to underdiagnosis. Persons with persistent respiratory symptoms should be encouraged to seek medical attention and return to a health care provider if symptoms persist or return despite initial treatment (*8*,*9*). Timely TB diagnoses save lives and prevent further community transmission.

The findings in this report are subject to at least two limitations. First, this analysis is limited to provisional TB surveillance data reported for 2020. In previous years, final case counts have not differed substantially from provisional data. However, although anecdotal information from reporting areas demonstrates that underreporting is not a major contributor to the reported decrease in TB incidence during 2020, underreporting from providers and underdiagnosis are possible. Second, denominators used to calculate incidence are based on estimated population numbers and might change slightly if population estimates are adjusted.

Further work is in progress to examine the causes of the steep decrease in reported TB cases. The extent of underdiagnosis will be explored by using external data sources of mortality, TB hospitalization, and anti-TB drug dispensation. Further analysis of laboratory data and conversations with clinical infection preventionists will help determine the extent of underreporting. In addition, changes in recent transmission will be examined by using isolate genotyping data. Identifying reversible causes of underdiagnosis or actual causes of an actual reduction in TB cases during 2020 will help identify effective public health responses. Supporting public health infrastructure for performing fundamental principles of TB control (e.g., case detection, contact tracing, and targeted testing and treatment for latent TB infection) is important. CDC remains committed to working with its public health partners to eliminate TB in the United States.

**Summary**

**What is already known about this topic?**

Tuberculosis (TB) incidence has decreased by an average of 2%–3% annually during the previous 10 years.

**What is added by this report?**

TB incidence during 2020 (2.2 cases per 100,000 persons) was 20% lower than that during 2019 (2.7 cases). The relative decrease in incidence was similar among U.S.-born and non–U.S.-born persons.

**What are the implications for public health practice?**

The steep decrease in TB incidence during the COVID-19 pandemic might be the result of reduced transmission and undetected cases. Health care providers should consider TB disease in patients with signs and symptoms consistent with TB, and the public should be encouraged to seek medical care when needed. Timely TB diagnoses save lives and prevent the spread of TB.

## Footnotes

* This report is limited to National Tuberculosis Surveillance System data verified as of February 17, 2021. Updated data will be available in CDC’s annual TB surveillance report later in 2021.

†
https://www.cdc.gov/tb/programs/rvct/instructionmanual.pdf

§
https://www.census.gov/data/tables/time-series/demo/popest/2010s-national-total.html

¶
https://www.census.gov/topics/population/race/about.html

**
https://www.census.gov/programs-surveys/cps.html

†† A person is considered U.S.-born if eligible for U.S. citizenship at birth, regardless of place of birth.

§§ Proportions were calculated excluding persons with missing race or ethnicity data.

¶¶ Small changes in case numbers or population size can lead to large relative changes because of the small size of this group.

***
https://www.cdc.gov/tb/education/professionaltools.htm
